# A Systemic Review on the Association Between Infertility and Sexual Dysfunction Among Women Utilizing Female Sexual Function Index as a Measuring Tool

**DOI:** 10.7759/cureus.16006

**Published:** 2021-06-28

**Authors:** Okelue E Okobi

**Affiliations:** 1 Family Medicine, Lakeside Medical Center, Belle Glade, USA

**Keywords:** infertility, sexual function, sexual dysfunction, women fsfi, female sexual function index, female factor infertility, female infertility, male factor infertility

## Abstract

The Center for Disease Control and Prevention describes infertility as the inability to conceive after one year or longer with adequate unprotected sex. Infertility affects both females and males, interfering with their everyday lives and significantly impacting their mental health. Sexual dysfunction is defined as an alteration of the sexual response cycle phases, preventing satisfaction during sexual activity. The prevalence of sexual dysfunction in the United States is high, with about 10%-52% among men and 25%-63% among women. Different scales can measure sexual satisfaction and double as a tool to diagnose sexual dysfunction. The Female Sexual Function Index (FSFI) is the gold standard for diagnosing sexual dysfunction in women. Overall, fertile women had a higher score on the FSFI than infertile women; however, both groups showed sexual dysfunctions even if the fertile group was classified as a mild disorder. The most common disorders were disorders of desire and lubrication. Desire and arousal dysfunction appeared more common in participants with secondary infertility, while lubrication dysfunctions were more common in older participants. In the future, it will be important to evaluate other factors that affect sexual function and fertility including mental health, male health, and couple factors.

## Introduction and background

Infertility is a common diagnosis in men and women. Couples are usually tested for infertility; however, women are more affected. Alterations in mental health can lead to depression, anxiety, and stress, which can translate into sexual dysfunction disorders; Nevertheless, studies show that infertility almost equally affects the sexual health of the couple. The Center for Disease Control and Prevention [1] describes infertility as the inability to conceive after adequate unprotected sexual intercourse for one year or more for women under 35 years and for six months or more for those over 35 years while men remain fertile well into their adulthood. Fertility in women is at its peak between teens and early thirties and subsequently decreases with age, with a more rapid decline after 35 years. For people in their 40s, the American Society of Reproductive Medicine says the average chance of pregnancy is around 5% each cycle. However, the number of people getting pregnant in their 40s is going up every year, largely due to the increasing uptake of assisted reproductive technology (ART) procedures. Infertility is classified as primary and secondary [2]. According to the World Health Organization [3], primary infertility is used for women with infertility who have never previously conceived. Secondary infertility is the inability to conceive in a couple who has previously conceived before; thus, it has multiple causes, varying among men and women. Causes of female infertility include ovulation disorders from polycystic ovarian syndrome, tubal blockage from previous genital tract infections, endometriosis or pelvic surgeries, hormonal disorders and imbalances, uterine factors from synechiae, submucous fibroids or polyp, congenital uterine anomalies, and others such as obesity, smoking, toxins, and unexplained causes.

Causes of male infertility include sperm abnormalities, previous pelvic surgeries such as herniorrhaphy or hydrocelectomy, metabolic diseases such as diabetes mellitus, medications for chronic illness, genetic factors, occupational and environmental factors, urological causes, and others such as smoking, drugs, and toxins.

Several studies have [4] shown a correlation between infertility, anxiety, and depression. In addition, adverse mental health can be of the primers for sexual dysfunction. Brotto et al. reported the interdependence of sexual function between partners and the psychological impact on at least one partner [5]. Sexual dysfunction refers to an alteration of any of the sexual response cycle phases, preventing satisfaction during sexual activity. The sexual response cycle has four stages: excitement (including arousal and desire), plateau, orgasm, and resolution. Men typically go through these phases in order, while women may not. According to statistics, sexual dysfunction is common among males and females; however, patients rarely discuss it openly because of the varying cultural sensitivities on the subject [6].

Classification of sexual dysfunction disorders

Sexual dysfunction disorders are commonly classified into five categories: desire disorders (including lack or loss of sexual desire, sexual aversion, excessive sexual drive), arousal disorders (failure for genital response), orgasm disorders (like orgasmic dysfunction, lack of sexual enjoyment, premature ejaculation), pain disorders (such as nonorganic dyspareunia, nonorganic vaginosis), and other disorders, (including paraphilia and gender identity disorders). The onset and context can also classify sexual dysfunction; they can be lifelong when they present as early onset of sexual functioning or acquired when developed after a period of normal functioning; they can be generalized when it is not limited to certain types of situations or partners or situational when it is limited to a problem or partner only [7]. Infertility and sexual dysfunction have almost similar or overlapping etiologies. Sexual dysfunction disorders can be caused by different factors, both physical and psychological causes. The physical causes that are notably medical conditions like endocrine disturbances, cardiovascular diseases, neurological disorders, kidney diseases or liver failure, substance abuse, some gynecological conditions, and some post pelvic infections, causing both sexual dysfunction and infertility (for example, hypothyroidism or hyperprolactinemia), may cause lack of sexual desire as well as difficulty in conceiving. Others are medications (such as antidepressants and antihypertensive medication), sexual dysfunction caused by infertility treatments, and iatrogenic causes. The psychological causes include stress, depression, anxiety, guilt, marital problems, past sexual trauma, and concerns about body image [6]. The prevalence of sexual dysfunction in the United States ranges from about 10%-52% among men and 25%-63% among women. This wide range of prevalence may be attributable to varying sensitivities on the subject and reluctance in the reportage [8]. Different scales can be used to measure sexual satisfaction as a tool to diagnose sexual dysfunction. There are generic scales for males and females like the Arizona Sexual Experience Scale, scales for males like the Index of Erectile Dysfunction, and scales for females like the Female Sexual Function Index (FSFI) [9].

For this analysis, we will be focusing on the FSFI as a tool for diagnosing sexual dysfunction in women from the eligible manuscripts from January 2011 to June 2021. Over the last 20 years, the FSFI has been considered a standard for measuring sexual function in women. Universally, it is the most commonly used scale, allowing for consistent and comparable results throughout independent studies. The FSFI is a self-report measure that evaluates 19 items to determine the sexual function of women and possible alterations in sexual desire, arousal, orgasm, pain, and satisfaction. The 19 items use a five-point Likert scale, with higher points indicating better sexual function [10]. To score the measure, the score of each domain is multiplied by a domain factor ratio to put all areas at a comparable level. The FSFI focuses on sexual activity within the last four weeks. If the woman has not been sexually active in that period of time, she will obtain a score of zero [11]. The score ranges from 0 to 36 [12]: 0-10 means severe sexual dysfunction, 11-16 means moderate sexual dysfunction, 17-25 means mild sexual dysfunction, and 26-30 means no sexual dysfunction. Previous studies have evaluated infertility and sexual function. The impact has been more pronounced in women compared to men [13]. In general, systemic reviews and meta-analysis report that women with infertility have higher sexual dysfunction when compared to fertile women [14]. The main limitation with this set of studies is the heterogeneity of the studies chosen. This meta-analysis will focus on the relationship between infertility with sexual dysfunction among women using FSFI as a measuring tool for sexual function. An earlier meta-analysis in 2017 by Mendonca and his group of researchers demonstrated the correlation between sexual dysfunction and infertility. This review attempts to add more recent literature searches beyond 2017.

Methodology

A systematic review of the medical database was performed. A search was done in PubMed, Medline, ScienceDirect, and other sources (Google Scholar) published between January 2011 and June 2021. The keywords used were infertility, sexual function, sexual dysfunction, women FSFI, and Female Sexual Function Index.

Inclusion Criteria

The inclusion criteria are studies focusing on sexual dysfunction and infertility, studies less than 10-years old, studies reporting their results using the FSFI scale, randomized control trials, controlled (non-randomized) studies, studies that included women without comorbidities as a cause of the sexual dysfunction and/or infertility, and studies including women that have not had sex within the past four weeks since this affects the validity of the FSFI.

Exclusion Criteria

Sexual dysfunction studies older than 2011 were excluded; sexual dysfunction studies that included women with comorbidities as a cause of their infertility were also excluded. Nineteen studies were identified in this search after the initial identification; following which 11 were excluded as they did not meet the inclusion criteria. The remaining eight studies were categorized based on their level of evidence using the Eccles-Mason categorization [15]. All studies were categorized as belonging to category I; information on the population used for the study and FSFI score was extracted and presented in tables.

## Review

**Figure 1:**
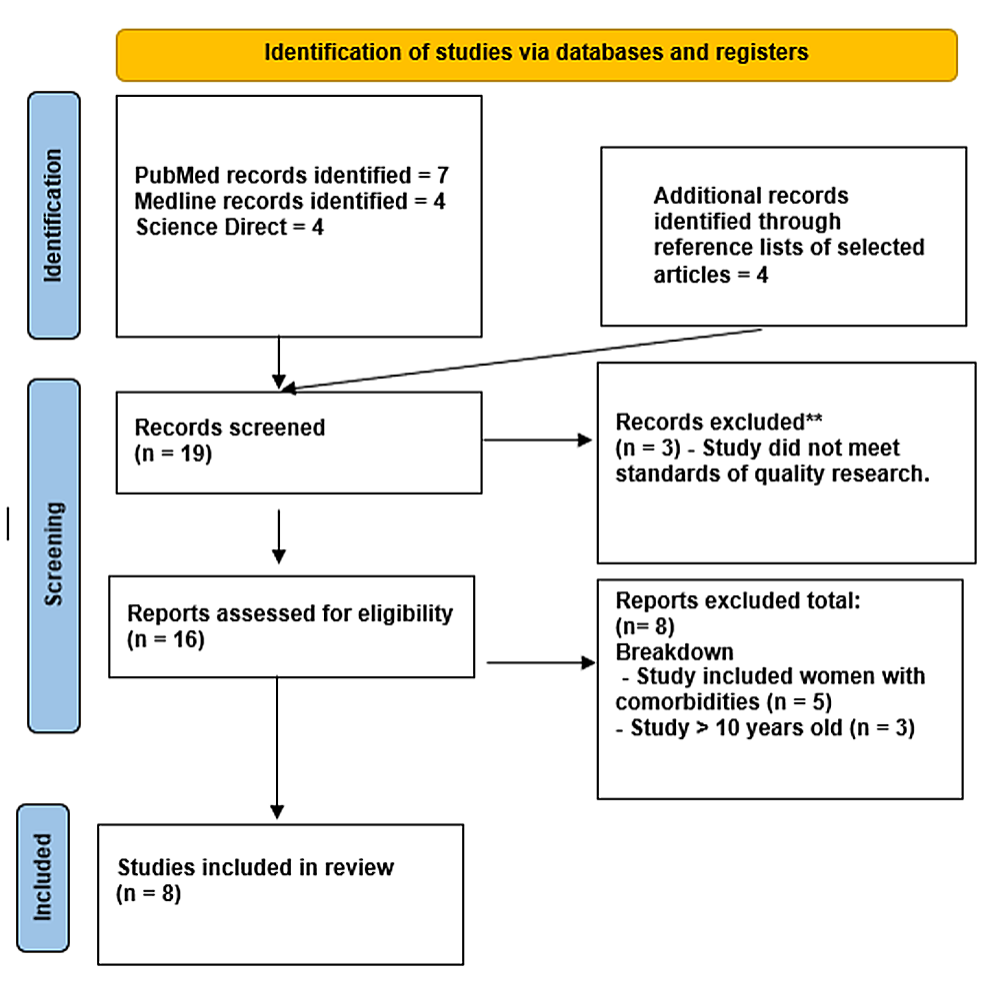
Flowsheet for study selection

The PRISMA flow diagram for sexual dysfunction and infertility systematic reviews is shown in Figure 1.

Results

Eight studies met our inclusion criteria; they reported infertile women against a control group. All the included studies used comparable populations in terms of the number of women evaluated and age group. These studies are listed below in Table 1.

**Table 1:** Population and age group per study

Study	Infertile and fertile women	Age in years
Tanha et al. [16]	191 Infertile 87 Fertile	32.4 ± 5.4
Marci et al. [12]	60 Infertile 52 Fertile	31.5 ± 9.35
Jamali et al. [17]	100 Infertile 100 Fertile	28.56 ± 5.72
Ashraf et al. [7]	172 Infertile 172 Fertile	31.74 ± 8.07
Emec et al. [18]	137 Infertile 142 Fertile	Not reported
Basirat et al. [10]	208 Infertile	Not reported
Mirblouk et al. [19]	147 Infertile 149 Fertile	30.61 ± 6.67
Aggarwa et al. [20]	125 Infertile 125 Fertile	33 ± 10.8

**Figure 2:**
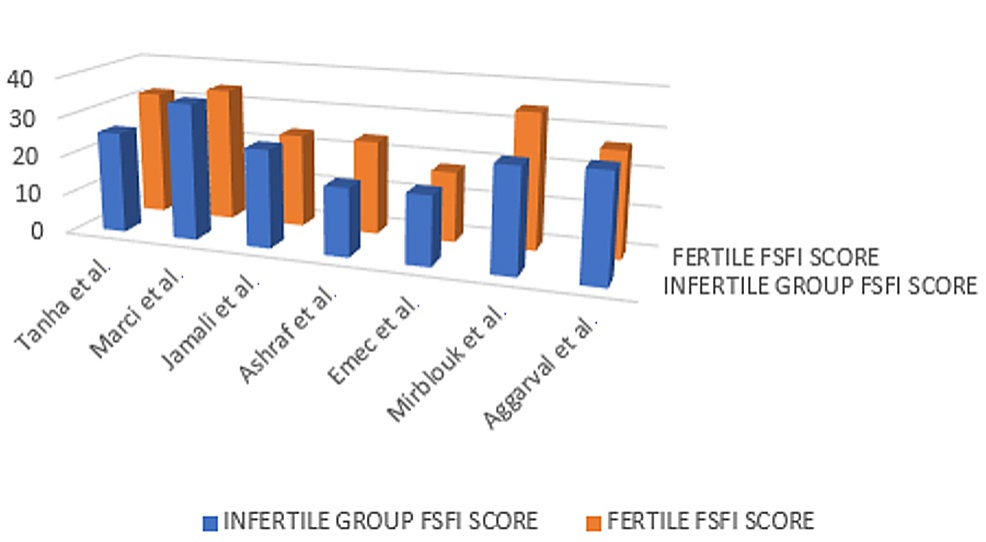
FSFI scores by study of infertile versus fertile women FSFI, Female Sexual Function Index

These literature reports associate values of FSFI below 26 to represent some degree of sexual dysfunction. The individual results of FSFI of each study are reported in Figure 2, comparing infertile versus fertile women.

**Figure 3:**
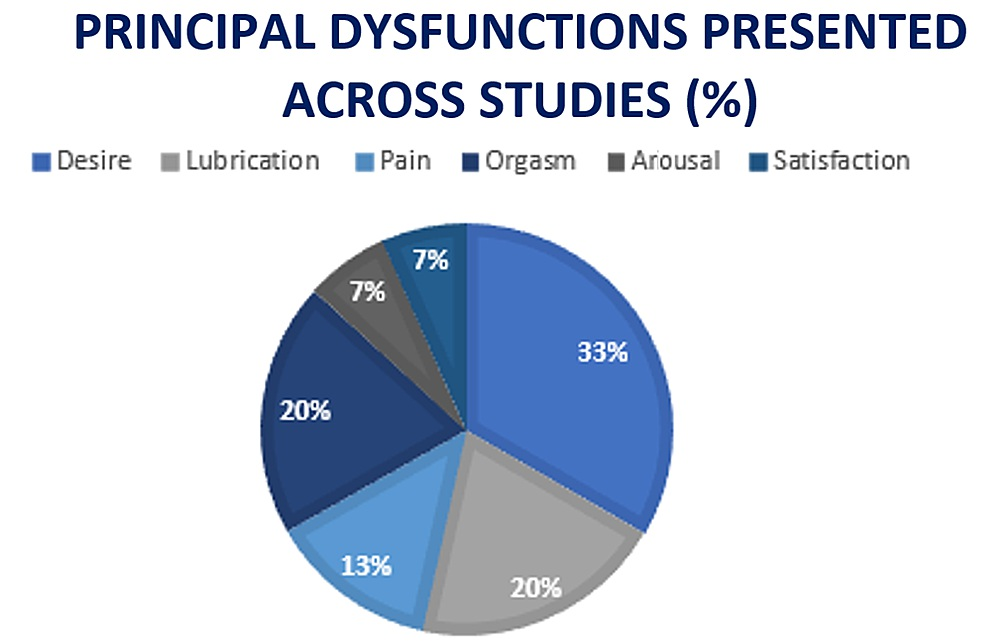
Principal sexual dysfunctions presented across studies

Overall, fertile women had a higher score on the FSFI than infertile women; however, both groups showed sexual dysfunctions. The fertile group's FSFI was classified as a milder disorder. Two studies provide us with the percentage of women who obtained scores for sexual dysfunction in both infertile and fertile groups; the Emec et al. study reported that 76.8% of the women without infertility problems and 78.8% of the women with infertility problems had sexual dysfunctions, and the difference between the groups was not statistically significant (p > 0.05) [18] and 63.67% infertile group vs. 43.65% in the fertile women for the study by Aggarwa et al. [20]. From the collated studies, FSFI categorizes sexual dysfunction in the breakdown below in Figure 3.

**Figure 4:**
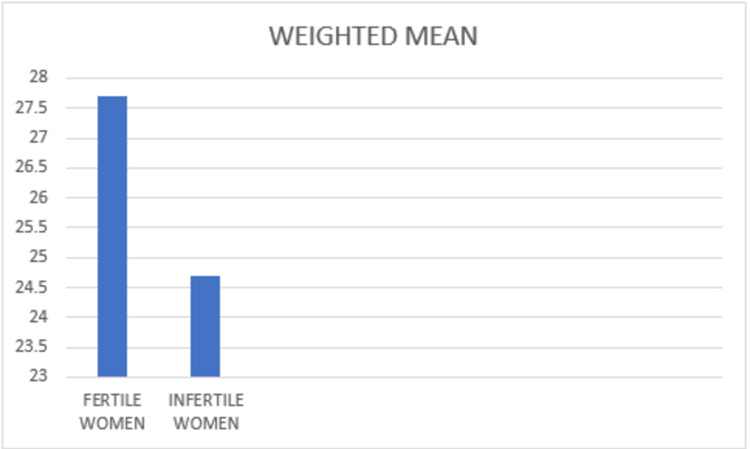
Weighted Mean

While finding supports that the most common disorders were dysfunctions of desire and lubrication, desire and arousal showed to be more common in participants with secondary infertility. In comparison, problems with lubrication were more common in older participants. The weighted mean of the studies was calculated and compared between infertile women and fertile women (Figure 4).

Discussion

Infertility [21] is a common diagnosis in gynecological outpatient clinics. For many couples, prolonged infertility can be severely distressing, affecting sexuality and the fundamental aspect of an individual’s life. Alterations in mental health can lead to depression, anxiety, and stress, which can translate into sexual dysfunction disorders. Nevertheless, studies have shown that infertility almost equally affects the sexual health of the couple [21]. Infertile couples have been known to suffer consequences, including psychosocial medical, economic, and cultural consequences, particularly in developing countries. The description of infertility is mutually exclusive for adequacy in sexual function, with adequacy in sexual intercourse, described as unprotected sexual intercourse between couples for at least three to four times a week, most weeks, for at least one year. Several reports of decreased sexual desire and frequency of intercourse within couples are due to various psychological, often unspoken, physical, and/or other factors. Several factors play a role in infertility; these factors include the male and female factors and the combined and other unknown factors. One common denominator between the male and the female factor is the inability to have adequate sexual intercourse. This inability to have sexual intercourse has been described in various literature to consist of stress factors, distance factors, the incompatibility factor, other factors, and other psychosocial factors, which all play a role in the inability to engage in sexual activity [22]. Understanding the role of these psychosocial stress factors may help in easing the scourge of infertility. Since many natural conceptions anchor on sexual intercourse and natural elements that contribute to conception, understanding these impeding psychosocial factors may help clarify the relationship between sexual dysfunction and the ability to conceive. Sexual dysfunction is also a relatively independent factor that exists between couples. The relationship between sexual dysfunction and infertility has been studied over the years, and several researchers have made several attempts to make a correlation between these two disorders. Sexual dysfunctions are characterized by disturbances in sexual desire and the psychophysiological changes associated with the sexual response cycle in men and women [8]. In addition, alterations in mental health can lead to depression, anxiety, and stress, which can translate into sexual dysfunction disorders; nevertheless, studies show that infertility almost equally affects the sexual health of the couple [21].

In a longitudinal study [23] to access the impact of partner coping in couples dealing with infertility, Danish men and women who were about to start a cycle of assisted reproduction treatment were followed for five years of unsuccessful treatments. Multilevel modeling using the actor-partner interdependence model was used to examine the couple as the unit of analysis; the result concluded that active and passive avoidance coping strategies were significantly related to increased personal, marital, and social distress at the individual and partner level. According to Jane Read [24], infertility may interact with an individual's sexual drive, desires, and other components of sexuality in many ways. These sexual problems may be caused or exacerbated by the diagnosis, investigation, and management of infertility or its associated variants, or the societal pressures on childlessness may also play a role. The medical management of a couple's difficulty in conceiving must include overt and transparent questioning about their psychosocial and sexual activities. The response to the inability to conceive triggers emotions (panic, despair, anger, and grief), further exacerbating the dysfunctional effects on sexual function. In addition, the stress of infertility and its treatment may cause sexual difficulties for both prospective patients. Coitus may be evaded, with several evasive patterns, perhaps, so that an individual or the partner is not reminded of the infertility problem. Furthermore, the associated counseling, medical consultations, and consequent fertility tests, such as semen samples, invasive procedures like hysterosalpingography, hormonal assays may result in a feeling of pressure to perform, adversely affecting sexual functions, deep pelvic muscle contractions and dyspareunia, erectile or ejaculatory ability; for some men, one or two failures during sex begins a vicious series of fear of failure, with anxiety driving further failures. Partners may also witness arousal difficulties because of distress and anxiety. Some people feel that their companion seems to want sexual intimacy only when there is a prospect of conception; for example, during the female ovulation periods, sexual activity may become a battleground for control, power, and the quest for a child [24].

In evaluating sexual dysfunctions, we used the FSFI because it is a very commonly used tool to assess sexual dysfunction and also a way to compare studies homogenously to determine any differences among studies that utilized combined scores and indexes to determine sexual function. Tanha et al. [16] evaluated infertility and sexual function in different cohorts in their study. They compared the FSFI score in infertile women, fertile women, and a control group. They noted a predominantly higher total score in the control group (32.1) than the infertile cohort (25.7), with a significant p-value of <0.001. They concluded that sexual dysfunction is more elevated in infertile women when compared to the rest of the cohorts. Similarly, Marci et al. [12] in a prospective study assessed infertility in couples and the relationship of sexual function in couples using various scales, including the FSFI. With a significant p-value < 0.005, across their multivariable endpoints, they concluded that infertile couples experience great stress on their sexual activity, especially in arousal and desire. Also, this correlative association was found in the Jamali et al. study [17]. Across the evaluated studies, most of them show that infertile women have some degree of sexual dysfunction with FSFI scores below 26. This is consistent with findings from previous literature [25,26]. One of the studies [17] found better sexual function among infertile women, but it concluded that these results were not significant after statistical analysis. The evaluated studies also show that fertile women also experience some degree of sexual dysfunction despite obtaining higher scores. The studies used for this meta-analysis were homogenous and comparable; they found a relevant correlation between infertility and sexual dysfunction with a p-value < 0.001; however, they evaluated essential sexual dysfunction and infertility aspects. In the future, it will be interesting for randomized control trials to incorporate scores for assessing mental health, couple’s sexual dysfunction, and studies focused only on men.

## Conclusions

Sexual dysfunction and infertility are common conditions; however, the relationship between them may be mutually exclusive, and in many circumstances, may not! However, where it exists, it can be detrimental to the affected people's mental health, compromising their sexual function and complicating its management. By evaluating independent studies, we tried to identify a correlation between sexual dysfunction and infertility and an association every clinician and patient needs when considering infertility management, especially when all other sexual dysfunctions or infertility factors have been excluded. In the future, designing studies to determine a more evidence-based association between sexual dysfunction and infertility will be helpful to patients and clinicians who deal with this dilemma. Also, the limited number of available randomized trials and uniformity in variables and measures may limit this analysis. A multicenter randomized control trial will be required to validate this relationship and overcome these limitations.
